# Pattern of the Divergence of Olfactory Receptor Genes during Tetrapod Evolution

**DOI:** 10.1371/journal.pone.0002385

**Published:** 2008-06-11

**Authors:** Takushi Kishida

**Affiliations:** Department of Zoology, Kyoto University, Kyoto, Japan; Ecole Normale Supérieure de Lyon, France

## Abstract

The olfactory receptor (OR) multigene family is responsible for the sense of smell in vertebrate species. OR genes are scattered widely in our chromosomes and constitute one of the largest gene families in eutherian genomes. Some previous studies revealed that eutherian OR genes diverged mainly during early mammalian evolution. However, the exact period when, and the ecological reason why eutherian ORs strongly diverged has remained unclear. In this study, I performed a strict data mining effort for marsupial opossum OR sequences and bootstrap analyses to estimate the periods of chromosomal migrations and gene duplications of OR genes during tetrapod evolution. The results indicate that chromosomal migrations occurred mainly during early vertebrate evolution before the monotreme-placental split, and that gene duplications occurred mainly during early mammalian evolution between the bird-mammal split and marsupial-placental split, coinciding with the reduction of opsin genes in primitive mammals. It could be thought that the previous chromosomal dispersal allowed the OR genes to subsequently expand easily, and the nocturnal adaptation of early mammals might have triggered the OR gene expansion.

## Introduction

Tetrapods can recognize various environmental odors using olfactory receptors (ORs). ORs belong to the superfamily of seven transmembrane G-protein coupled receptors (GPCRs) and consist of one of the largest multigene families in vertebrate genomes [1]–[3]. The number of functional OR genes varies greatly among terrestrial tetrapod species, ranging from approximately 80 genes in chickens to 1200–1600 genes in rats [4]–[6]. It has been reported that the number of functional OR genes appears to parallel the reliance on the sense of smell in a species. The fraction of OR pseudogenes seems to have increased in the primate lineage leading to humans [7], suggesting a reduced dependence on olfaction as a result of the acquisition of full trichromatic color vision [8]. Large scale degeneration of OR genes is found in cetaceans, which have secondarily adapted to a marine habitat and have lost or greatly reduced their sense of smell acquired in terrestrial environments [9]. These findings suggest that the number of intact OR genes reflects the ability of odor recognition in tetrapod species.

OR genes are scattered widely in placental mammalian chromosomes. For example, OR genes can be found on every human chromosome except for chromosomes 20 and Y [10]. Using the limited data available in 2001 [11], Glusman et al. estimated that OR genes had migrated from chromosome 11 to other chromosomal regions mainly before 310 MYA, before the mammal-bird split. They also indicated that OR genes were evolutionarily relatively stable between the mammal-bird split and placental-marsupial split during vertebrate evolution.

Recently, an SWS2 class opsin gene, which encodes one of the four spectrally distinct classes of vertebrate cone pigment and has never been found in marsupial or placental mammals, was found in a monotreme platypus, suggesting that placental mammals lost their sense of color vision gradually during early mammalian evolution between the mammal-bird split and the placental-marsupial split [12]. As mentioned above, primates have compensated for their reduced sense of smell by acquisition of trichromatic color vision, and it could also be hypothesized that primitive mammals compensated for their reduced sense of color vision by enlargement of the size of their OR repertoires. This hypothesis suggests that the size of our OR repertoires expanded mainly in the period between the mammal-bird split and the placental-marsupial split, and that the placental-marsupial last common ancestor (LCA) had acquired a large number of ORs. However, Glusman et al. estimated that OR genes expanded mainly after the placental-marsupial split [11].

In this study, I have performed a data mining effort for marsupial opossum OR genes strictly and estimated the evolutionary change of chromosomal migration and the size of OR repertoires in the tetrapod lineage leading to modern Euarchontoglires using 6 genome-sequenced tetrapod species, including opossum, and following the method designed by Suga et al. [13]–[14] for estimating the periods of gene migrations and duplications.

## Results and Discussion

Table 1 shows the number of opossum OR genes identified in this study (available as supporting Text S1). The total number of OR genes generally agreed with other independent reports [5]–[6]. The pseudogene fraction might have been underestimated because a number of pseudogenes would be included in the partial intact genes. In addition to the opossum OR gene database, previously reported OR gene databases for 5 tetrapods (Table 2) were used in this study. Partial sequences and pseudogenes were excluded from further analyses because their inclusion would have sharply reduced the alignment regions. The chromosomal distribution of mouse OR genes, obtained from the Trask Laboratory mouse OR gene database (http://www.fhere.org/science/labs/trask/OR/), is shown in Table S1.

**Table 1:** Number of opossum OR genes identified in this study.

No. of genes identified	Complete sequences		Partial sequences	
	intact genes	pseudogenes	intact genes	pseudogenes
1548	953	77	452	66

Note: An OR sequence which lacks clear transmembrane domains and/or a complete open reading frame is defined as a pseudogene. An OR sequence beginning with an initiation codon, ending with a termination codon and longer than 810 bp is defined as a complete sequence.

**Table 2:** Number of OR genes of each species analyzed in this study. Partial sequences and pseudogenes were excluded.

Species		No. of genes analyzed	References
Amphibia	Frog	477	[22]
Aves	Chicken	103	[22]
Monotremata	Platypus	260 (30/230)*	[31]
Marsupialia	Opossum	953 (192/761)*	This study
Laurasiatheria	Dog	645 (131/514)*	HORDE database**
Euarchontoglires	Mouse	1120 (126/994)*	Trask Laboratory mouse OR gene database***

* Numbers in parenthesis are (no. of class I genes/no. of class II genes)

** [23], http://bioportal.weizmann.ac.il/HORDE/, downloaded on 1 May 2007.

*** [32], http://www.fhere.org/science/labs/trask/OR/, downloaded on 31 May 2007.

**Figure 1:**
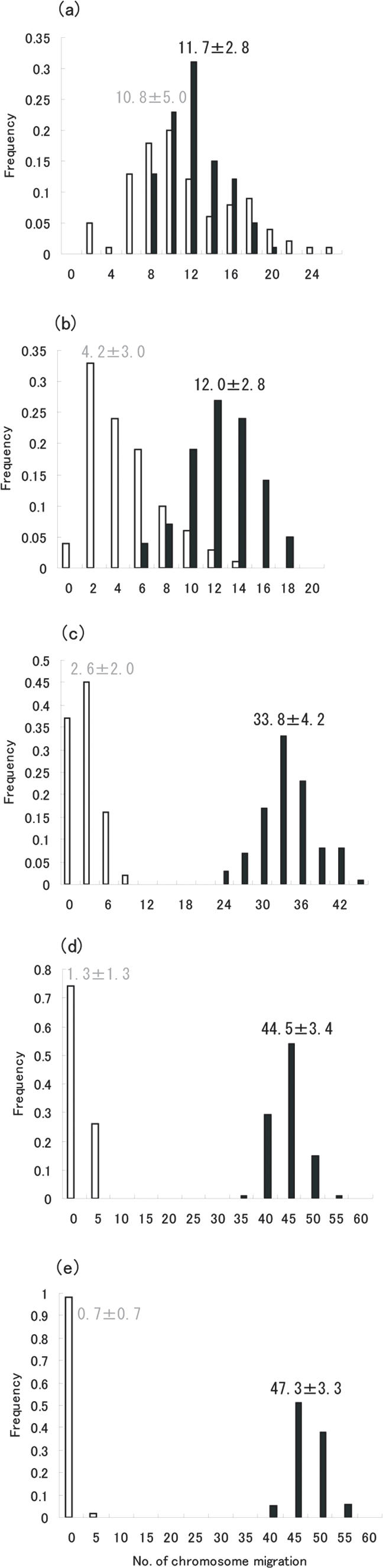
The number of chromosomal migrations of mouse OR genes before (black bars) and after (white bars) the Amphibia-Mammalia (a), Aves-Mammalia (b), Monotremata-Placentalia (c), Marsupialia-Placentalia (d) and Laurasiatheria-Euarchontoglires (e) split, respectively based on the bootstrap analysis. The distribution of the number of chromosomal migrations was calculated by repeating the bootstrap resampling procedure [28] 100 times and by constructing the phylogenetic tree for each resampling procedure based on the neighbor-joining method [29]. Mean±standard deviation is shown in the figure. In mammals, only class II ORs were taken into account because all class I ORs are located in the same chromosomal region [11].

**Figure 2:**
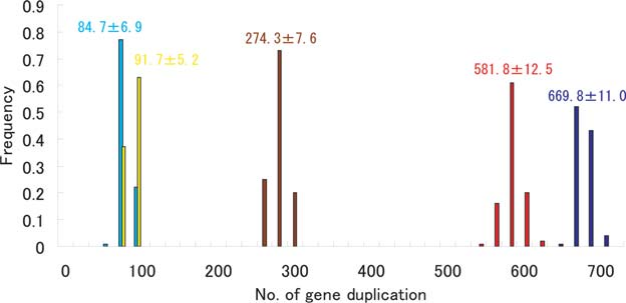
The number of gene duplications before the Amphibia-Mammalia (light blue bars), birds-Mammalia (yellow bars), Monotremata-Placentalia (brown bars), Marsupialia-Placentalia (red bars) and Laurasiatheria-Euarchontoglires (blue bars) split, based on the bootstrap analysis. Mean±standard deviation is shown in the figure. The detailed data for mammals are shown in Table S2.

Bootstrap analyses were performed by the standard procedure with 100 resamplings, modified from the method designed by Suga et al. [13]–[14] (Text S2), in order to calculate the number of chromosomal migrations (Fig. 1) and the number of gene duplications (Fig. 2). Fig. 1 indicates that chromosomal migrations occurred mainly during early vertebrate evolution before the monotreme-placental split. In contrast, regarding gene duplications, Fig. 2 indicates that OR genes were duplicated mainly in the period between the mammal-bird split and the placental-marsupial split. These results suggest that chromosomal dispersal occurred ahead of gene expansion.

Vertebrates are known to have developed well-established tetrachromatic color vision before the fish-tetrapod split [15]. The vertebrate tetrachromatic color vision relies on four spectrally distinct classes of cone pigment encoded by distinct opsin genes: SWS1, SWS2, Rh2 and LWS classes [16]. It has been reported that placental mammals lost the SWS2 and Rh2 classes after the bird-mammal split and now retain only the LWS and SWS1 classes, and this loss is thought to have occurred because of the nocturnal lifestyle of primitive mammals [16]. Some Australian marsupials are suggested to have evolved trichromatic color vision [17]. As yet, however, in spite of substantial efforts, no SWS2 or Rh2 opsin genes have been identified in any marsupial genomes [16], which strongly suggests that mammals had degenerated into having dichromatic color vision before the placental-marsupial split. Recently, an SWS2 class opsin gene was found in the platypus genome [12], indicating that the SWS2 class opsin gene was lost in the placental mammalian lineage after the placental-monotreme split. On the other hand, no Rh2 class opsin gene was found in the platypus genome [12], which suggests that the Rh2 class might have been lost before the placental-monotreme split. Considering all these things, it could be concluded that mammals lost their sense of color vision gradually between the mammal-bird split and the placental-marsupial split because of nocturnal adaptation. In this study, Fig. 2 indicates that a large-scale duplication of OR genes occurred in the placental mammalian lineage between the mammal-bird split and the placental-marsupial split, and it appears that the expansion of OR genes coincided with the reduction of opsin genes. A nocturnal lifestyle would have required a well-established sense of smell regardless of the sense of color vision. It can be said metaphorically, that the chromosomal scattering of OR genes would have been a fuse for an explosive, and the nocturnal adaptation might have triggered the OR gene expansion.

**Figure 3:**
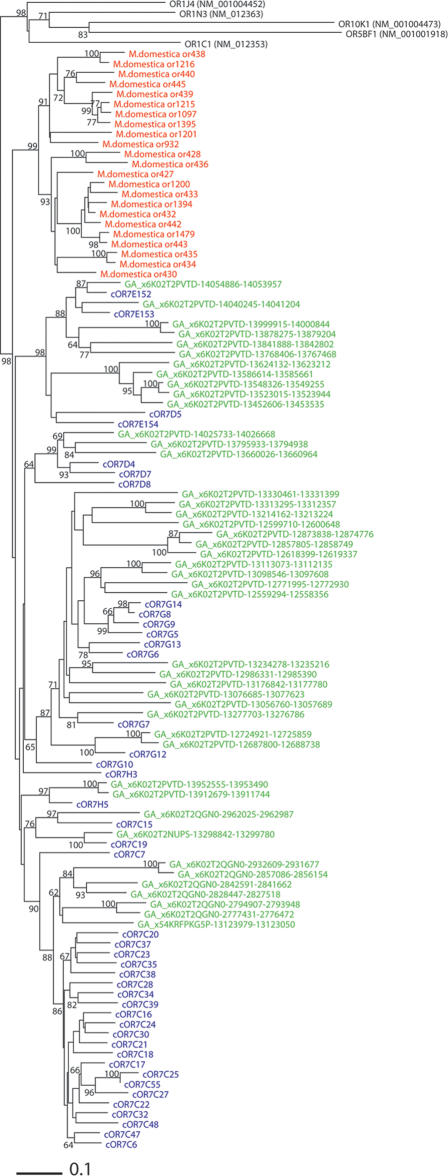
A phylogenetic tree of an OR gene subgroup called family 7. The tree was constructed by the neighbor-joining method [29], based on the Poisson correction distance [30] matrices. OTUs written using red fonts indicate opossum ORs, blue fonts indicate dog ORs and green fonts indicate mouse ORs. Five human ORs belonging to other subgroups were used as outgroups. Bootstrap values were obtained by 1000 resamplings, and the values >60% are shown.

However, phylogenetic analysis suggested that one subgroup of OR genes called family 7 [18], which comprises the largest subgroup in the human OR gene repertoire [11], diverged after the placental-marsupial split (Fig. 3). Interestingly, the family 7 subgroup contains some receptors which are thought to have become necessary very recently during mammalian evolution, such as the human OR7D4 receptor, which is activated only by androstenone or androstadienone pheromones [19]. Further studies of the family 7 subgroup could be expected to reveal some interesting aspects of modern eutherian evolution.

Finally, the estimated numbers of OR genes possessed by our ancestors are shown in Table 3. The estimation method is detailed in the ‘Materials and methods’ section. The estimated gene numbers indicate that OR genes diverged gradually, with the major divergence occurring during early mammalian evolution, between the mammal-bird split and the placental-marsupial split. The estimated numbers in the monotreme-placental LCA and the marsupial-placental LCA (which would be underestimated, as explained in ‘Materials and methods’) are much larger compared to those in a previous report [6], perhaps due to the following facts: (i) the previous method did not consider the number of genes which were lost in both lineages after speciation ( = *bc*/*a*, according to Eq. 9'), and (ii) the previous method adopted the condensed tree method [30] for evaluating the reliability, which must underestimate the number of LCA genes because ambiguous subtrees would not be considered in the condensed trees. The other OR databases are also analyzed and the results essentially support the main conclusion of this study (Table S4).

**Table 3:** Number of OR genes in LCA estimated using Eq. 9 and based on the bootstrap analysis.

	Estimated no. of genes
Amphibia-Mammalia LCA	113.1±11.4
Aves-Mammalia LCA	101.8±8.3
Monotremata-Placentalia LCA	327.1±13.1
Marsupialia-Placentalia LCA	667.2±19.4
Laurasiatheria-Euarchontoglires LCA	737.0±15.8

The distribution of the number of Type-A, B and C subtrees was calculated by repeating the bootstrap resampling procedure based on the neighbor-joining method. Mean±standard deviation is shown. The details are shown in Table S3.

## Materials and Methods

### 1. Collecting opossum OR repertoire

The opossum OR gene repertoire was constructed from the opossum draft genome sequence database downloaded from the Ensembl trace server (ftp.ensembl.org/pub/traces/monodelphis_domestica) on 20/DEC/2004 (ver. e!27). For each sequence, regions with low-quality scores (quality value<10, according to the quality files) were cut off to get reliable data. The TFASTY program [20] was carried out against these genome sequences to identify OR coding regions using human, mouse and zebrafish known OR gene sequences as queries. As a result, 8410 OR related sequences were obtained.

In order to merge sequences which come from the same OR gene, two sources of intralocus variation must be taken into account: interallelic variation and sequencing error. I tried four conservative stringency values, 98.0%, 98.5%, 99.0% and 99.5%. Except for the value of 99.5%, there was at least one group of three sequences which did not satisfy the transitive law, i.e. seq. A = seq. B and seq. B = seq. C, but seq. A ≠ seq. C. Therefore, I opted for a conservative stringency value of 99.5% with >100bp overlap to minimize erroneous clone merging. Finally, the sequences were aligned with known OR genes to identify the amino acid coding regions. All sequences were searched against the entire GenBank using the BLAST program [21] to ensure that their best three hits were known ORs.

### 2. Phylogenetic analyses

It has been reported that large mammalian OR genes can clearly be classified into two subfamilies (class I and class II) based on the sequence similarity, while non-mammalian OR genes cannot be as easily classified as mammalian ORs because of their wide diversity [3], [22]. In this study, mammalian OR sequences were divided into two subfamilies and each subfamily was analyzed independently to obtain more accurate results. Dog ORs were classified according to the classification in the HORDE database ([23], http://bioportal.weizmann.ac.il/HORDE/). Platypus, opossum and mouse OR sequences were searched against the HORDE#42 human OR database using the FASTA3 program [24] and classified into class I or II subfamilies according to their most similar human sequences.

Deduced amino acid sequences of OR genes in compared species were aligned using the MAFFT program [25] with manual adjustments. Positions with alignment gaps were excluded from further analyses. The root of the tree of vertebrate OR genes is difficult to determine because even the closest non-OR GPCR gene is too divergent to provide accurate root information. In this study, an amphioxus GPCR gene (amphi-GPCR1, GenBank accession no. AB182635) was used as an outgroup, as suggested by Satoh [26]. The trees of mammalian class I OR genes were rooted by a class II human OR gene (OR2T4, GenBank accession no. NM_001004696), and class II trees by a class I human OR gene (OR51M1, GenBank accession no. NM_001004756). The aligned sequence data analyzed in this study are available as supporting Text S3, S4, S5, S6, S7, S8, S9, and Text S10.

### 3. Estimation of the number of ancestral OR genes

**Figure 4:**
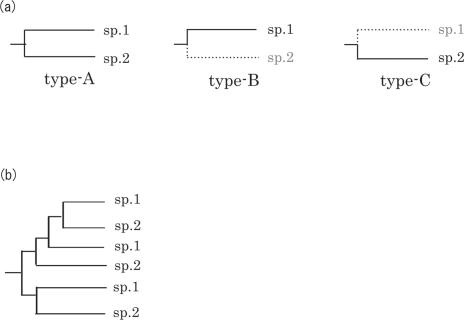
Three phylogenetic subtree types of a phylogenetic tree consisting of 2 species (sp.1 and sp.2). Genes derived from intraspecific duplications are considered to be one gene. Type-A indicates that a gene is found in both sp.1 and sp.2 lineages. Type-B indicates that orthologous genes of a gene of sp.1 are not found in sp.2. Type-C indicates that orthologous genes of a gene of sp.2 are not found in sp.1. (a) An imaginary tree of a multigene family in two species (sp.1 and sp.2). It can be resolved into two type-A subtrees, one type-B subtree and one type-C subtree.

Every multigene phylogenetic tree consisting of two species (sp.1 and sp.2) can be resolved into three types of phylogenetic subtrees, if genes derived from intraspecific duplications are considered to be one gene (Fig. 4(a)). For example, the imaginary tree shown in Fig. 4(b) can be resolved into 2 type-A subtrees, 1 type-B subtree and 1 type-C subtree. Here, the number of subtrees is denoted by *a* for type-A, *b* for type-B and *c* for type-C. The set of sp.1-sp.2 LCA genes is denoted by G_0_. Subsets of G_0_ passed on to sp.1 or sp.2 are denoted by G_1_ and G_2_. Then, the following equations hold (|G| is the number of elements of the set G, and G_1_^c^ is the complement of G_1_):
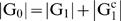
(1)

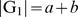
(2)

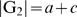
(3)

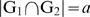
(4)

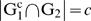
(5)


On the assumption that genes in the lineages leading to sp.1 or sp.2 evolve independently, namely, subset G_1_ and G_2_ are independent from each other, the following equation is obtained:
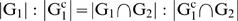
(6)


If *x* is defined as the number of LCA genes ( = |G_0_|), the following equation is derived from Eq. 1 and Eq. 2:
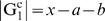
(7)


Then Eq. 6 can be expressed in terms of *a*, *b*, *c* and *x* using Eq. 2, Eq. 4, Eq. 5 and Eq. 7:
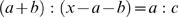
(8)


Finally, the following equation is obtained by solving the equation Eq. 8:
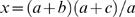
(9)


Eq. 9 means that the number of LCA genes can be estimated by counting the number of type-A, B and C subtrees. Eq. 9 can be expanded as follows:
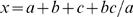
(9')


The value *a*+*b*+*c* stands for the number of G_0_ genes which are remaining in G_1_ and/or G_2_ genomes, and according to Eq. 9', the value *bc*/*a* is revealed to stand for the estimated number of G_0_ genes which were lost in both G_1_ and G_2_ lineages.

Some sources of potential errors, however, should be noted in the estimation of the value of *x* in Eq. 9. Alternative gene loss in sp.1 and sp.2 between two adjacent subtrees, concerted evolution [27] or some positive correlations between G_1_ and G_2_ might lead to underestimation of the number of LCA genes.

## Supporting Information

Table S1 (0.02 MB PDF)

Table S2 (0.04 MB PDF)

Table S3 (0.04 MB PDF)

Table S4 (0.07 MB PDF)

Text S1 Opossum OR database obtained in this study
(1.45 MB TXT)

Text S2 Supporting materials and methods
(0.02 MB TXT)

Text S3 The OR sequences aligned between frog and mouse
(1.13 MB TXT)

Text S4 The OR sequences aligned between chicken and mouse
(0.94 MB TXT)

Text S5 The class I OR sequences aligned between platypus and mouse
(0.07 MB TXT)

Text S6 The class II OR sequences aligned between platypus and mouse
(0.84 MB TXT)

Text S7 The class I OR sequences aligned between opossum and mouse
(0.14 MB TXT)

Text S8 The class II OR sequences aligned between opossum and mouse
(0.99 MB TXT)

Text S9 The class I OR sequences aligned between dog and mouse
(0.14 MB TXT)

Text S10 The class II OR sequences aligned between dog and mouse
(1.24 MB TXT)
