# Supplementary material for: Pattern of the Divergence of Olfactory Receptor Genes during Tetrapod Evolution
Source: PLoS One. 2008 Jun 11;3(6):e2385. doi: 10.1371/journal.pone.0002385 (PMC2435047; doi:10.1371/journal.pone.0002385)
Supplement: Table S1 — (0.02 MB PDF) [file pone.0002385.s001.pdf]

Table S1. Chromosomal distribution of mouse OR genes, according to the Trask Laboratory mouse OR gene database (http://www.fhere.org/science/labs/trask/OR/).

Chromosomal loci	No. of genes analyzed
1D	13
1H	13
2B-C	29
2E	260
4B	11
6B	22
6F	5
7E	229 (126)*
8B-C	5
9A	134
10B-C	9
10D	54
11B	78
13A	12
13C	1
14A	2
15F	9
16B	42
17C	51
19C	60
XA	6
Unknown	75
Total	1120 (126)*

Note *Number in parenthesis is the number of class I genes
