# Supplementary material for: Pattern of the Divergence of Olfactory Receptor Genes during Tetrapod Evolution
Source: PLoS One. 2008 Jun 11;3(6):e2385. doi: 10.1371/journal.pone.0002385 (PMC2435047; doi:10.1371/journal.pone.0002385)
Supplement: Table S2 — (0.04 MB PDF) [file pone.0002385.s002.pdf]

Table S2. The number of class I and II duplications used in Fig. 2. The number of class I and II gene duplications before the platypus-mouse, opossum-mouse and dog-mouse split, respectively based on the bootstrap analyses are indicated. Average ± standard deviation is shown.

	Platypus-Mouse	Opossum-Mouse	Dog-Mouse
Class I	47.5±3.0	134.2±3.6	115.8±2.3
Class II	226.8±6.4	447.7±11.8	553.9±10.5
