# Supplementary material for: Pattern of the Divergence of Olfactory Receptor Genes during Tetrapod Evolution
Source: PLoS One. 2008 Jun 11;3(6):e2385. doi: 10.1371/journal.pone.0002385 (PMC2435047; doi:10.1371/journal.pone.0002385)
Supplement: Table S3 — (0.04 MB PDF) [file pone.0002385.s003.pdf]

Table S3. The detailed dataset of Table 3.

		no. of type-A subtrees	no. of type-B subtrees	no. of type-C subtrees	no. of estimated genes
Amphibia-Mammalia LCA					
	sp.1: mouse				
	sp.2: frog	28.0±2.8	37.4±5.3	20.3±2.4	113.2±11.4
Aves-Mammalia LCA					
	sp.1: mouse				
	sp.2: chicken	23.6±1.5	65.9±4.9	3.2±1.5	101.8±8.3
Monotremata-Placentalia LCA					
	sp.1: mouse				
	sp.2: platypus	(16.9±0.8/76.9±3.2)	(29.5±2.9/121.2±5.6)	(2.1±0.9/29.8±3.2)	(52.1±3.6/275.0±12.1)
Marsupialia-Placentalia LCA					
	sp.1: mouse				
	sp.2: opossum	(69.7±1.7/207.0±3.4)	(23.3±2.0/138.4±7.0)	(42.2±3.0/103.3±6.7)	(149.3±5.4/517.9±18.6)
Laurasiatheria-Euarchontoglires LCA					
	sp.1: mouse				
	sp.2: dog	(73.9±1.5/278.5±3.4)	(19.6±1.8/190.0±7.2)	(23.3±1.8/86.4±5.5)	(123.0±3.1/614.0±15.3)

Note: Numbers in parentheses mean (class I / class II).
