# Supplementary material for: Pattern of the Divergence of Olfactory Receptor Genes during Tetrapod Evolution
Source: PLoS One. 2008 Jun 11;3(6):e2385. doi: 10.1371/journal.pone.0002385 (PMC2435047; doi:10.1371/journal.pone.0002385)
Supplement: Table S4 — (0.07 MB PDF) [file pone.0002385.s004.pdf]

Table S4-1. Counted number of type-A, B, C subtrees and estimated number of LCA genes using data shown in Table S4-2. Distributions of the number of type-A, B, C subtrees and estimated LCA genes were calculated by repeating the bootstrap resampling procedure 100 times and by inferring the phylogenetic tree for each resampling procedure based on the neighbor-joining method. Each value represents mean±standard deviation.

	sp.1<br>sp.2	tetrapods<br>fishes	mammals and birds<br>amphibians	mammals<br>birds	placentals<br>marsupials	Laurasiatheria<br>Euarchontoglires
Class I	No. of type-A subtrees	*	*	*	84.9±1.7	73.8±1.5
	No. of type-B subtrees	*	*	*	33.1±3.3	23.0±2.1
	No. of type-C subtrees	*	*	*	39.7±2.7	19.9±2.0
	Estimated no. of genes possessed by LCA	*	*	*	173.3±6.2	122.9±3.8
Class II	No. of type-A subtrees	*	*	*	260.5±3.9	278.7±3.8
	No. of type-B subtrees	*	*	*	204.3±11.2	85.8±5.3
	No. of type-C subtrees	*	*	*	109.3±7.6	189.6±7.9
	Estimated no. of genes possessed by LCA	*	*	*	660.0±26.2	612.6±15.9
Total	No. of type-A subtrees	5.1±0.4	29.7±2.9	23.9±1.4	345.4±4.2	352.4±4.1
	No. of type-B subtrees	11.3±3.9	44.0±5.8	83.7±6.4	237.4±12.3	108.8±6.0
	No. of type-C subtrees	10.5±2.2	17.9±2.5	3.4±1.7	149.0±8.6	209.5±8.1
	Estimated no. of genes possessed by LCA	50.2±13.9	118.4±11.7	123.3±10.5	833.2±28.4	735.5±16.9

Table S4-2. References for the OR gene databases of each species analyzed

Species		no. of genes analyzed	references
Osteichthyes	zebrafish	102	[22]
	pufferfish	47	[22]
Amphibia	frog	477	[22]
Aves	chicken	103	[22]
Marsupialia	opossum	862 (172/690)[a]	HORDE database
Laurasiatheria	dog	645 (131/514)[a]	HORDE database
Euarchontoglires	mouse	1120 (126/994)[a]	Trask Laboratory mouse OR gene database

Note [a] Numbers in parentheses means (no. of class I genes / no. of class II genes).
