# Supplementary material for: Pattern of the Divergence of Olfactory Receptor Genes during Tetrapod Evolution
Source: PLoS One. 2008 Jun 11;3(6):e2385. doi: 10.1371/journal.pone.0002385 (PMC2435047; doi:10.1371/journal.pone.0002385)
Supplement: Text S2 — Supporting materials and methods (0.02 MB TXT) [file pone.0002385.s006.pdf]

Text S2. Supporting Materials and Methods

Estimating the number of gene duplications and migrations

The OR gene databases analyzed in this study are shown in Table 2. Pseudogenes and short partial sequences (less than 810bp) are excluded because their inclusion would have sharply reduced the alignment regions. The aligned sequences are available as supporting text S3 (frog – mouse), S4 (chicken – mouse), S5 (platypus – mouse, class I), S6 (platypus – mouse, Class II), S7 (opossum – mouse, class I), S8 (opossum – mouse, class II), S9 (dog – mouse, class I) and S10 (dog – mouse, class II).

The number of gene duplications is counted based on the phylogenetic trees. For example, two consecutive gene duplications before the sp.1–sp.2 split are counted in an imaginary tree shown in Figure S1 (see below). The distribution of the number of gene duplications is calculated by repeating the bootstrap resampling procedure [28] 100 times and by inferring the phylogenetic tree for each resampling procedure using the neighbor-joining method [29] based on the Poisson correction distance matrices [30]. The histograms of the distributions are shown in Figure 2.

The number of gene migrations is also counted based on the phylogenetic trees and the distributions are calculated as mentioned above. In this study, the number of gene migrations is defined as the number of gene duplications that gave rise to different chromosomal regions.

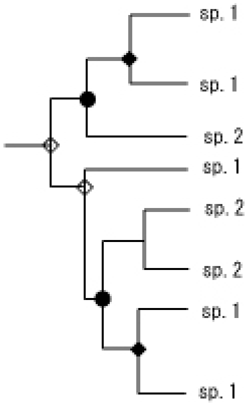

Figure S1. An imaginary tree of a multigene family in two species (sp.1 and sp.2). Filled circles indicate the sp.1 – sp.2 split; open (filled) rhombi indicate gene duplications that antedate (postdate) the sp.1 – sp.2 split in the lineage leading to sp.1.

The number of ancestral OR genes using other data sources

The supporting analyses on OR genes were carried out between fishes (zebrafish and pufferfish) – tetrapods (frog and mouse), amphibians (frog) – non-amphibian tetrapods (chicken and mouse), birds (chicken) – mammals (opossum and mouse), marsupials (opossum) – placentals (mouse and dog) and Laurasiatheria (dog) – Euarchontoglires (mouse). The number of type-A, B and C subtrees (see Materials and Method section) and the estimated numbers of OR genes in the LCA of these species are shown in Table S4. Platypuses are excluded from the supporting analyses because their OR repertoires are highly degenerated [31]. The results are essentially support the main conclusion of this study. The number of genes in the marsupial-placental LCA is higher compared to the overall results, and this is simply because the sum of the number of type-A, B and C subtrees is increased by using the much larger number of OTUs as placental ORs.
